# Lipidomic Profiling of Milk Enables Identification of Novel Biomarkers for Subclinical Ketosis in Dairy Cows

**DOI:** 10.3390/foods15173117

**Published:** 2026-09-02

**Authors:** Zhiqian Liu, Vilnis Ezernieks, Joanne E. Hemsworth, Coralie M. Reich, Carolyn R. Bath, Monique J. Berkhout, Muhammad S. Tahir, Leah C. Marett, Amanda J. Chamberlain, Mike E. Goddard, Ruidong Xiang, Simone J. Rochfort

**Affiliations:** 1 Centre for AgriBiosciences, Agriculture Victoria, 5 Ring Road, Bundoora, VIC 3083, Australia; vilnis.ezernieks@agriculture.vic.gov.au (V.E.); joanne.hemsworth@agriculture.vic.gov.au (J.E.H.); coralie.reich@agriculture.vic.gov.au (C.M.R.); carolyn.bath@agriculture.vic.gov.au (C.R.B.); sajid.tahir@agriculture.vic.gov.au (M.S.T.); amanda.chamberlain@agriculture.vic.gov.au (A.J.C.); mike.goddard@agriculture.vic.gov.au (M.E.G.); ruidong.xiang@agriculture.vic.gov.au (R.X.); simone.rochfort@agriculture.vic.gov.au (S.J.R.); 2 School of Applied Systems Biology, La Trobe University, Bundoora, VIC 3083, Australia; monique.berkhout@agriculture.vic.gov.au (M.J.B.); leah.marett@agriculture.vic.gov.au (L.C.M.); 3 Ellinbank Centre, Agriculture Victoria, Ellinbank, VIC 3821, Australia; 4 School of Agriculture and Food, Faculty of Veterinary and Agricultural Sciences, The University of Melbourne, Melbourne, VIC 3010, Australia

**Keywords:** cows, subclinical ketosis, milk, fatty acids, lipidomic profiling, biomarkers

## Abstract

The current clinical test for ketosis in dairy cows, which is based on measuring blood β-hydroxybutyrate (BHB) concentrations, is an invasive procedure that impacts herd welfare. Alternative biomarkers, especially non-invasive ones, are urgently needed for subclinical ketosis (SCK) diagnosis. To address this, we examined the milk lipidomic profiles of healthy and SCK cows from a research dairy farm over two consecutive years. A total of 125 polar lipid species were quantified in milk samples using a targeted liquid chromatography–mass spectrometry (LC-MS) approach. Chemometric analysis identified four potential biomarkers (PC 28:0, PC 29:0, PC 30:1 and PC 31:0), which met the thresholds for *p* value (<0.0004) and fold change (FC > 2). Global fatty acid (FA) profiling by gas chromatography–flame ionization detection (GC-FID) after transesterification of all lipids revealed that the levels of C8:0, C10:0, C12:0, C14:0, C15:0 and C18:1t11 were significantly suppressed in the SCK group (15–52% lower compared to the control group, *p* < 0.01), and C15:0 and C18:1t11 were the most promising markers for SCK in dairy cows at the FA level (43–50% reduction compared to the control group, *p* < 0.0001). Tandem LC-MS and FA data generated by GC demonstrated that SCK also caused significant changes in regio-isomer and double bond isomer composition of a model phospholipid molecule (PC 34:1) and a model triglyceride molecule TAG 52:2; these fine-structural alterations could also be explored for discovering novel SCK biomarkers in dairy cows. The PLS-DA predictive model built from lipidomic data showed excellent accuracy (>95%) in predicting cows with SCK. All of the promising biomarkers identified and the predictive model built in this study need further validation using larger SCK cohorts from different herds.

## 1. Introduction

In the dairy industry, maintaining cow health is critical for optimizing milk yield and ensuring animal welfare. One prevalent metabolic disorder affecting dairy cows worldwide is ketosis, a condition characterized by elevated levels of ketone bodies in the blood due to excessive mobilization of body fat [1]. This metabolic disturbance often occurs in early lactation, a period when cows experience an imbalance between reduced dry matter intake and high energy requirements for milk production [2]. Ketosis can lead to reduced milk yield, impaired reproductive performance, and increased susceptibility to other diseases, ultimately impacting farm profitability and animal welfare [3,4].

Classic methods for detecting ketosis include clinical signs such as reduced milk yield, lethargy, decreased appetite, and a distinctive sweet or acetone-like odour on the breath [2]. These observable symptoms can indicate potential ketosis but are not definitive on their own. The gold standard method for diagnosing ketosis involves measuring the concentration of ketone bodies in blood [5]. Ketone bodies, primarily beta-hydroxybutyrate (BHB), acetoacetate, and acetone, are intermediates of FA metabolism that accumulate in the bloodstream during ketosis [6]. Blood tests for BHB are widely used because elevated levels (above 3.0 mmol/L) are a reliable indicator of ketosis, and portable blood ketone metres can provide quick on-farm results, aiding timely diagnosis and management of the condition [7].

Despite being effective for identifying and managing ketosis, BHB-based blood tests are invasive and costly. Milk BHB monitoring offers a low-cost and non-invasive alternative, but the lower sensitivity and specificity of milk BHB tests as compared to blood BHB hamper their widespread adoption as definitive diagnostic tool for SCK [8,9]. Many attempts were also made to predict blood BHB using milk mid-infrared spectroscopy (MIR) spectra, yielding moderate prediction coefficients (R^2^: 0.5–0.6) [10,11]. Extensive validation is still required before this technology can be implemented as diagnostic tools for individual animals.

Ketosis is a metabolic disease primarily caused by negative energy balance, resulting in impaired metabolic pathways, including hepatic gluconeogenesis, lipolysis, β-oxidation and ketogenesis [12,13]. As a result, significant changes in lipid composition and even lipid structure may be detectable in the body fluids of ketotic or even SCK cows. Recent advances in sophisticated analytical techniques, especially LC-MS, not only allow the facile detection of hundreds of lipid signatures in biological samples but also enable lipid structures (such as FA regio-position and double bond location) to be revealed [14].

Milk FA composition is prone to influence by both genetic factor and cow’s diets [15,16], but much less is known about the impact of SCK condition on milk de novo FA and rumen bacteria-derived FA (such as FA C15:0) [17]. In addition, milk contains hundreds of phospholipids [18], which are known to be signalling molecules [19], which may be sensitive to SCK condition. We hypothesize that SCK in postpartum dairy cows would induce a significant shift in both FA and the phospholipid profile of milk, and that sensitive and specific lipid biomarkers could be detected in the milk of SCK cows.

In this study, we aimed to (1) identify potential lipid biomarkers for SCK by FA profiling and targeted lipidomic profiling of milk from healthy and SCK cows, and (2) build and validate predictive models for healthy and SCK cow classification based on milk lipidomic data. In addition, lipid structural alterations, especially FA regio-isomer composition of representative phospholipid and triglyceride (TAG) molecules, induced by SCK conditions, were investigated using a tandem LC-MS approach.

## 2. Materials and Methods

### 2.1. Milk Samples and Chemicals

This research was conducted at the Agriculture Victoria Research Ellinbank SmartFarm in Victoria, Australia (38°14′ S, 145°56′ E). Milk samples (about 50 mL) were collected during morning milking from a cohort of 149 and 225 spring-calving (July–September) Holstein–Friesian cows in 2022 and 2023, respectively, at the early lactation stage (5–14 DIM). Each cohort comprised both primiparous heifers and multiparous cows (age: 2–11 years; parity: 1–9; average daily milk production across the lactation: 33 L). Cows that were sampled in 2022 were not sampled again in 2023. The large and heterogenous cohorts aimed to cover the inter-cow variation in response to postpartum metabolic adaptation. Milk samples were transported to the laboratory on ice and frozen immediately at −80 °C until analysis. Blood samples were collected immediately after morning milking from each cow and the serum BHB was determined by a ChemWell Analyzer [20]. All procedures were conducted in accordance with the Australian Code of Practice for the Care and Use of Animals for Scientific Purposes. Approval to conduct the experiment was obtained from the DEECA Agricultural Research and Extension Animal Ethics Committee (approval code: AEC 2022-04; approval date: 11 May 2022). Given that the blood BHB thresholds for SCK diagnosis in the literature range between 1.2 and 1.4 mmol/L [21], we used a serum BHB < 0.8 and >1.3 thresholds for selecting a subset of 45 healthy (control) and 45 SCK cows, respectively.

Regio-pure standards PC 16:0/18:1 and PC 18:1/16:0 (for PC 34:1), and OPO and OOP (for TAG 52:2) were purchased from Avanti Lipids (Alabaster, AL, USA). Fatty acid methyl ester (FAME) standard mix was sourced from Sigma-Aldrich (St. Louis, MO, USA). Ammonium formate (mobile phase additive) was of analytical grade (Sigma-Aldrich). Solvents used for milk lipid extraction and mobile phase preparation were of HPLC or LC-MS grade and were from Fisher Scientific (Pittsburgh, PA, USA) (methanol, isopropanol and 0.1% formic acid in acetonitrile), Sigma-Aldrich (chloroform and butanol) and Merck (Darmstadt, Germany) (acetonitrile).

### 2.2. FA Profiling of Milk Fat

For FA profiling of milk lipids, direct acid-catalyzed methylation method (without lipid extraction step) was adopted. Briefly, 100 µL of milk was mixed with 2.8 mL of 3% H_2_SO_4_ in methanol and incubated at 70 °C for 3 h. Quantification of FA methyl esters (FAMEs) was subsequently carried out by GC-FID following the detailed protocol described previously [22].

### 2.3. Targeted Lipidomic Profiling of Milk by LC-MS

Milk lipids were extracted using a one-phase method reported by Liu et al. [23]. Briefly, 50 µL of milk was mixed with 0.95 mL of butanol/methanol/chloroform (3:5:4, *v*/*v*/*v*) in a 2 mL Eppendorf tube; lipids were extracted by vortex at full speed for 2 min, followed by centrifugation for 15 min (at 13,000 *g*). The supernatant was used for lipidomic analysis by LC-MS. A pooled QC sample was prepared by mixing equal proportions of milk samples from 15 control and 15 SCK cows, and the lipids were extracted following the same procedure.

The LC separation of milk lipids was achieved on a Vanquish UHPLC system (Thermo Fisher Scientific, Waltham, MA, USA) with a HILIC column (150 × 4.6 mm, 2.6 µm, Phenomenex) maintained at 30 °C. The mobile phase was composed of 10 mM ammonium formate (A) and acetonitrile containing 0.1% formic acid (B). The gradient elution was performed by a linear increase in mobile phase A from 5 to 50% over 10 min with a flowrate of 0.5 mL/min. The injection volume was 4 µL.

A Q Exactive MS analyser (Thermo Fisher Scientific) operated simultaneously in positive and negative mode at a resolution of 70,000 was used for the quantification of targeted lipid species. The list of targeted lipid species was selected (based on their content) from the comprehensive milk lipid inventory published previously [18]. All individual samples were analyzed in a randomized order. The MS response fluctuation was monitored by analyzing the pooled QC sample after every 10 samples. The relative abundance (peak area) of all lipid species was used to identify biomarkers via chemometric analysis and construct classification models.

### 2.4. Determination of Regio-Isomer Composition of Milk Lipids by LC-MS/MS

The LC separation of milk lipids was conducted by an Eclipse XDB-C8 column (100 × 2.1 mm, 3.5 µm, Agilent Technologies, Santa Clara, CA, USA) on the Vanquish UHPLC system. The column compartment was maintained at 50 °C. The mobile phase was made up of water/acetonitrile (40:60, *v*/*v*) containing 10 mM ammonium formate (A) and acetonitrile/isopropanol (10:90, *v*/*v*) containing 10 mM ammonium formate (B). The gradient elution was performed by a linear increase in mobile phase B from 32 to 97% over 21 min with a flowrate of 0.25 mL/min. The injection volume was 4 µL.

An Orbitrap Elite mass spectrometer (Thermo Fisher Scientific) was employed to conduct parent ion (MS) and product ion (MS2) scans. The MS analyser was operated only in positive ion mode (4.2 kV) with a full scan (100–1200 *m*/*z*) at a resolution of 60,000, followed by data-dependent MS2 scan (in CID mode at 35 eV). The regio-isomer composition of PC 16:0/18:1 vs. PC 18:1/16:0 and TAG OPO vs. TAG OOP was determined using the intensity of diagnostic product ions and calibration curves generated with various proportions of regio-pure standard mix, as reported in detail previously [24,25].

### 2.5. Data Analysis

For FAME data generated by GC-FID, statistical comparisons between the healthy (control) and SCK groups were conducted using Student’s *t*-test (Microsoft Excel 365). Chemometric analysis of targeted lipidomic data for the identification of potential lipid biomarkers was carried out with MetaboAnalyst 6.0 (http://www.metaboanalyst.ca) [26]. The one-factor statistical module was adopted for PLS-DA analysis and feature visualization (by VIP score plot and volcano plot). Prior to analysis, the raw data were normalized by a pooled QC sample, Log transformed and auto-scaled to equalize the weight of all lipid features. Predictive models for SCK in dairy cows based on all lipidomic data were built with MATLAB 2024a (MathWorks, Natick, MA, USA) and PLS-Toolbox 9.3.1 (Eigenvector, Manson, WA, USA). The first model was built using 2022 data, and its prediction performance was tested on 2023 data. The second model was built with the combined data matrix of 2022 and 2023 split into calibration and prediction datasets using the Kennard–Stone algorithm with Euclidean distance measures, retaining 80% of the data for calibration. Both X and Y blocks were auto-scaled. Cross-validation used the Venetian blinds method with 10 splits and blind thickness = 1. The resultant model was tested for its predictive power on the remaining 20% of individual cows.

## 3. Results

### 3.1. Comparison of Milk FA Profile Between SCK and Healthy Cows

A total of 20 major FAs were quantified in milk samples from 21 (2022 cohort) and 24 (2023 cohort) SCK cows and an equal number of healthy (control) cows. The results were expressed as a percent of total FA. Despite discrepancies observed for a few FAs (e.g., C16:0, C17:0-iso and C18:3n3), most FAs followed a consistent pattern of response to SCK condition over two years. For example, C16:1, C18:0 and C18:1c9 were always increased (by 14–45%), whereas C8:0, C10:0, C12:0, C15:0, C18:1t11 and C18:2c9t11 were invariably decreased in the SCK group (by 15–52%) (Table 1). Among the differentially expressed species, C15:0 and C18:1t11 displayed very low *p*-values combined with high percentage changes (43–50% reduction), suggesting that these two FAs are the most promising markers for SCK in dairy cows at the FA level (Table 1).

### 3.2. Comparison of Milk Lipid Profile Between SCK and Healthy Cows (2022)

Biochemical analysis data of serum collected on the same day allowed us to identify 21 SCK cows (BHB > 1.3) from the 2022 cohort and the same number of control cows (BHB < 0.8) were randomly selected from the cohort. Targeted lipidomic profiling of milk samples by HILIC-MS enabled reliable quantification of 125 polar lipids belonging to 10 classes (PI, PE-P, PE, LacCer, PC, PC-P, LPC, PS, SM and AcylCar). The detailed structures of these milk polar lipids were all described in our earlier report [18].

**Figure 1:**
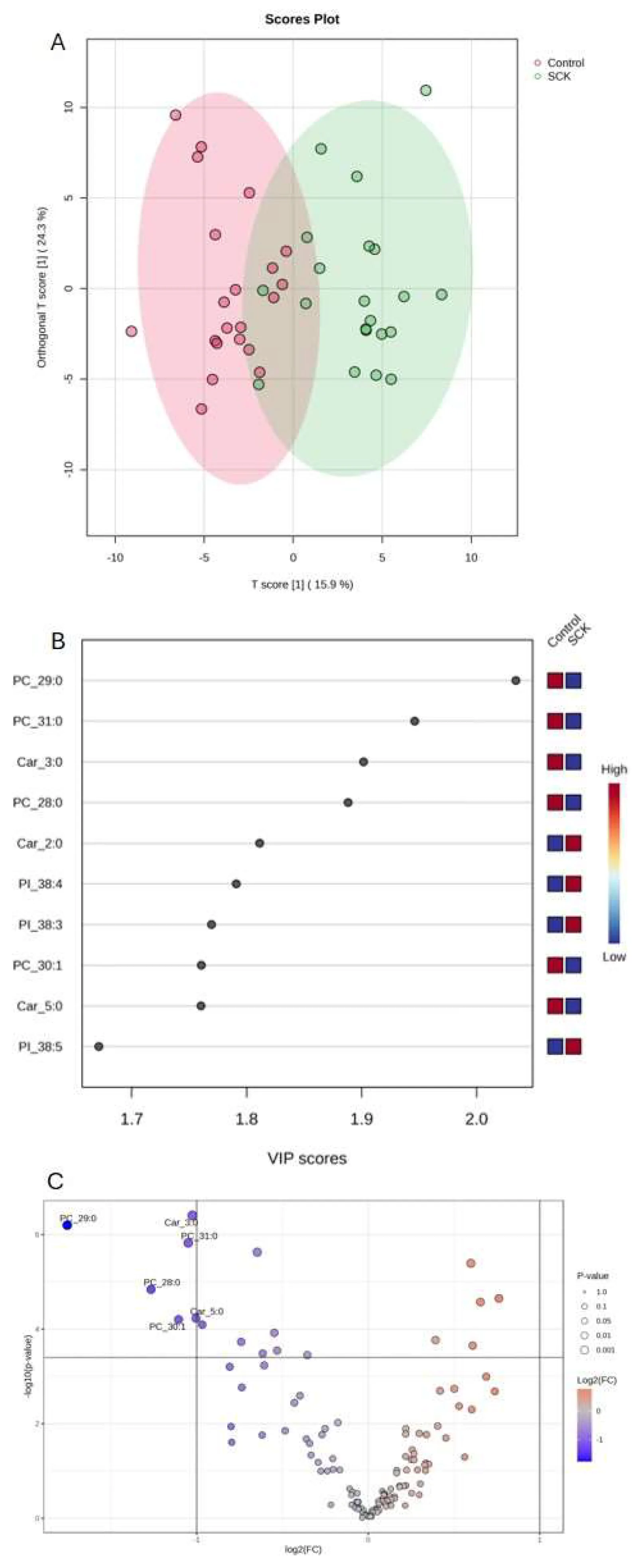
OPLS-DA plot of the control (*n* = 21) and SCK groups (*n* = 21) of 2022 cohort based on milk lipid profile (**A**); the top 10 most differential lipid species ranked by VIP score (**B**); and the volcano plot showing significantly down-regulated species based on fold change (SCK/control > 2) and *p* value (<0.0004) (**C**).

When the data matrix was subjected to Orthogonal Partial Least Squares Discriminant Analysis (OPLS-DA), two distinct clusters corresponding to the control and SCK groups were observed despite a few outliers in each group (Figure 1A), suggesting that SCK condition induced a significant shift in the overall milk lipidome. The top 10 most differential lipid species and their VIP (Variable Importance in Projection) scores are shown in Figure 1B; among them, four lipids were up-regulated (Car 2:0, PI 38:3, PI 38:4 and PI 38:5) and six were down-regulated (PC 30:1, PC 31:0, PC 28:0, PC 29:0, Car 3:0 and Car 5:0) in the SCK group. When both *p* value (<0.0004, Bonferroni-corrected significance level) and a fold change threshold (FC > 2) were set, only six lipids (PC 28:0, PC 29:0, PC 31:0, PC 30:1, Car 3:0 and Car 5:0) met both criteria (Figure 1C). All six lipids were downregulated in the SCK group, and all were included in the list of the top 10 most differential species identified based on VIP score.

### 3.3. Comparison of Milk Lipid Profile Between SCK and Healthy Cows (2023)

**Figure 2:**
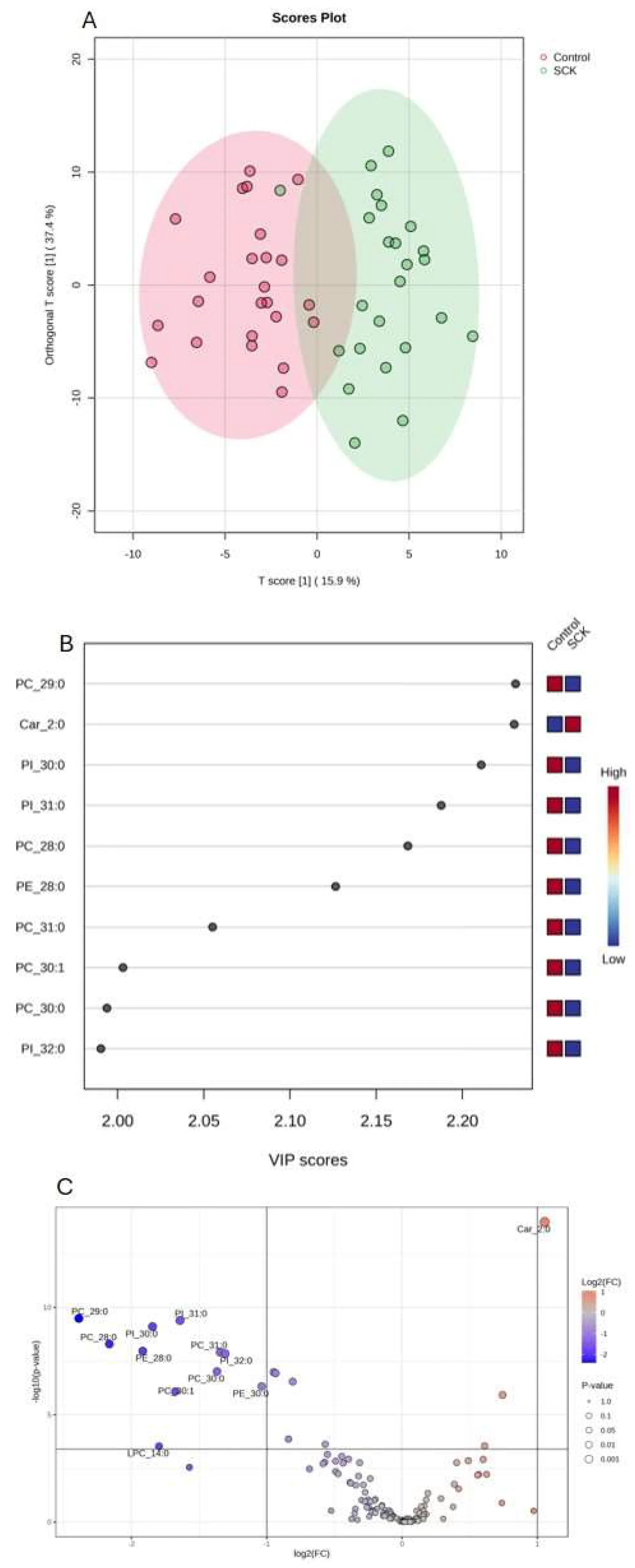
OPLS-DA plot of the control (*n* = 24) and SCK groups (*n* = 24) of 2023 cohort based on milk lipid profile (**A**); the top 10 most differential lipid species ranked by VIP score (**B**); and the volcano plot showing significantly down- and up-regulated species based on fold change (SCK/control > 2) and *p* value (<0.0004) (**C**).

A total of 24 SCK cows and 24 control cows were selected from the 2023 cohort. OPLS-DA analysis again revealed a quasi-complete separation of the control and SCK groups based on the abundance of the 125 polar lipids (Figure 2A). The top 10 lipid features contributing to the separation are shown in Figure 2B; among them, one species (Car 2:0) was up-regulated, while the remaining nine species (PC 28:0, PC 29:0, PC 30:0, PC 30:1, PC 31:0, PE 28:0, PI 30:0, PI 31:0 and PI 32:0) were downregulated in SCK cows (Figure 2B). When the same FC and *p* value thresholds as described previously were applied, Car 2:0 remained the only up-regulated lipid, whereas the total number of down-regulated species increased to 11. In addition to the top 10 lipids identified by VIP score, PE 30:0 and LPC 14:0 also met the criteria (Figure 2C).

When the results of the 2022 and 2023 cohorts were compared, four polar lipid species, namely PC 28:0, PC 29:0, PC 30:1 and PC 31:0, satisfied the criteria of FC and *p* value for two consecutive years.

### 3.4. Lipid Structural Change Induced by SCK Conditions

The potential structural change in milk lipids caused by SCK was examined only in the 2022 cohort, using the relative abundance of diagnostic ions acquired by tandem RP-LC-MS. The regio-isomeric composition was investigated for one representative polar lipid PC 16:0-18:1 and one TAG species TAG 16:0-18:1-18:1. The double bond (DB) locational isomer ratio was examined for the most abundant unsaturated FA of milk C18:1 based on GC-FID data.

**Figure 3:**
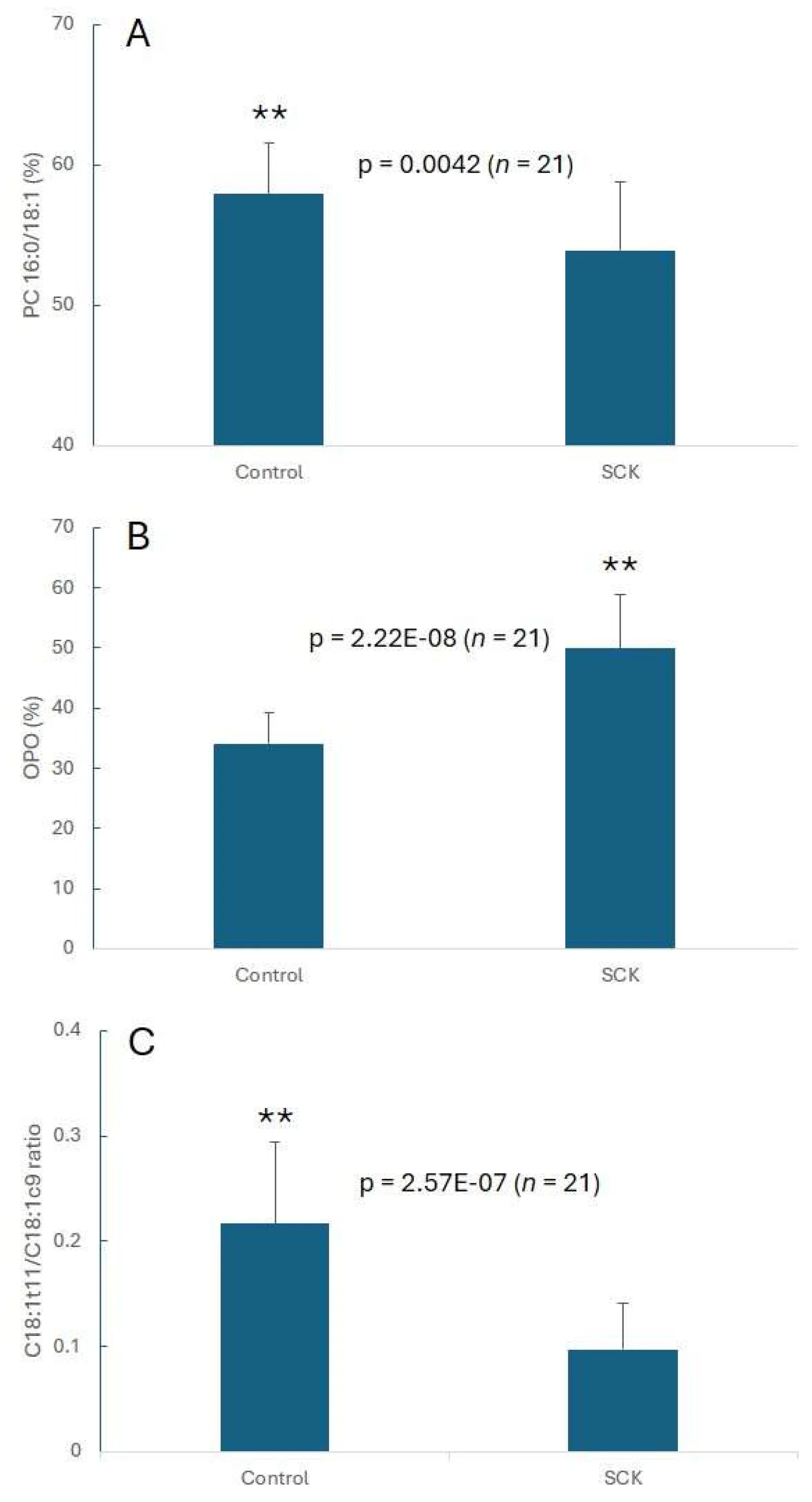
Regio-isomer proportion (%) of PC 16:0/18:1 (**A**), TAG OPO (**B**), and double bond location isomer ratio (C18:1t11 vs. C18:1c9) (**C**) in the milk of control and SCK cows (2022 cohort). ** *p* < 0.01.

SCK in dairy cows significantly reduced the ratio of PC 16:0/18:1 vs. PC 18:1/16:0 (Figure 3A) but significantly increased the proportion of TAG OPO (oleic acid/palmitic acid/oleic acid) vs. TAG OOP (oleic acid/oleic acid/palmitic acid) in milk lipids (Figure 3B). Due to the significant decrease in C18:1t11 and concomitant increase in C18:1c9, the ratio of these two DB locational isomers was significantly reduced in SCK cows (Figure 3C).

### 3.5. Prediction of SCK in Dairy Cows Based on Milk Lipidomic Data

**Figure 4:**
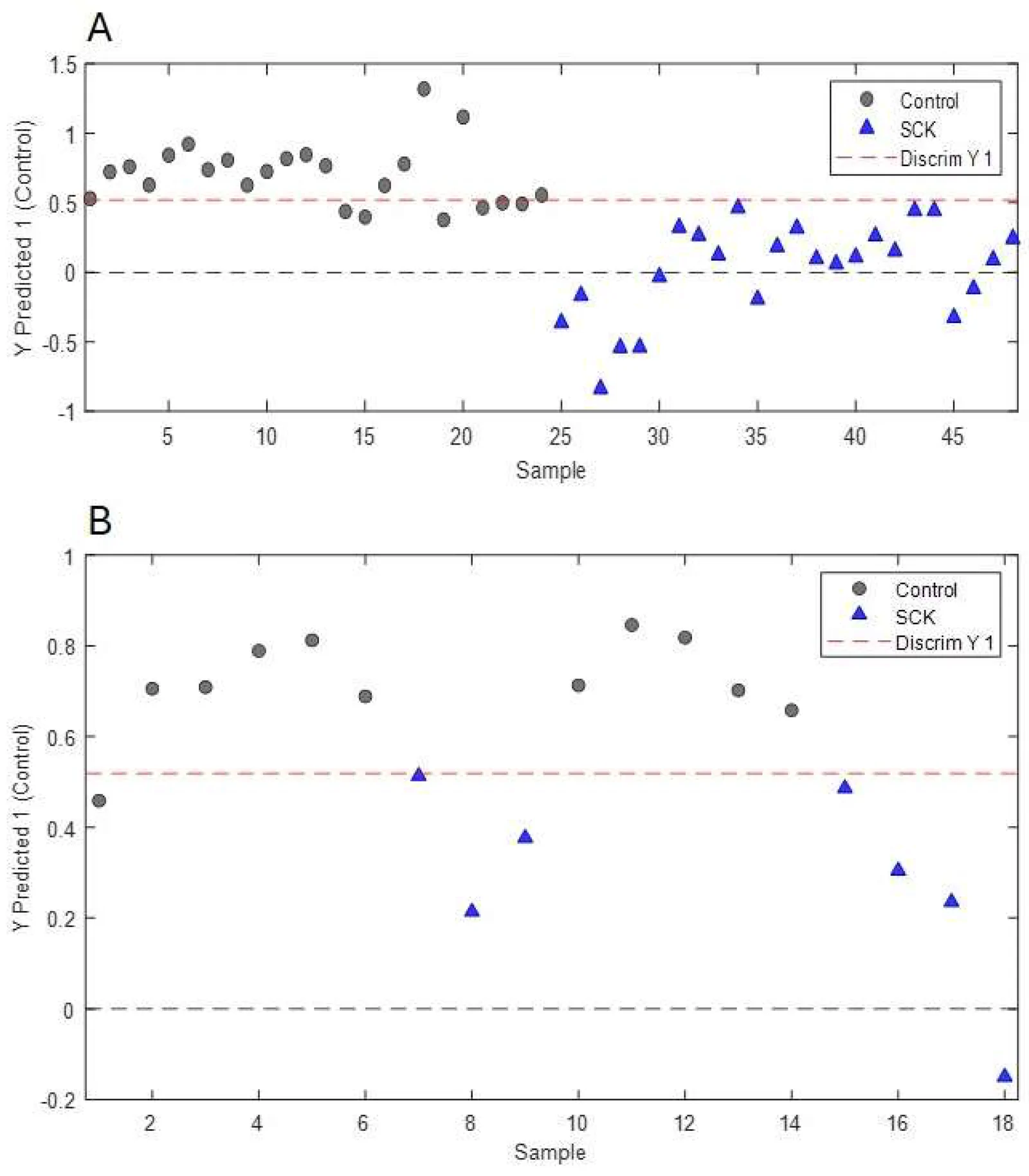
Performance of PLS-DA prediction models. (**A**): prediction of 2023 cohort using model built with 2022 data; (**B**): prediction of the remaining 20% cows using model built with 80% of cows combined from 2022 and 2023 cohorts.

First model built with 2022 data displayed a calibration and validation error of 7.1 and 9.5% with sensitivity and specificity (cross validation) between 0.86 and 0.95. When this model was applied to 2023 data, 42 out of 48 cows (87.5%) were correctly predicted (Figure 4B). Due to the limited number of SCK cows in the herd in any single year, the combined data of 2022 and 2023 were used to build a composite model for predicting SCK in dairy cows. The PLS-DA model was built with 80% of the data with the remaining data used to test the model. The model gave calibration and cross validation errors of 4.5–7% with sensitivity and specificity (cross validation) ≥ 0.91 (two latent variables). This model allowed accurate prediction of 17 out of 18 cows (Figure 4B). The prediction error was 4.5%, with sensitivity and specificity of 1.0 and 0.91, respectively.

## 4. Discussion

Given that ketosis is one of the most common and costly diseases in dairy herds [27], and the invasive nature of monitoring blood BHB, many attempts have been made to identify biomarkers from milk [28,29,30,31,32]. While milk MIR spectra-based predictive models could serve as a high-throughput, herd-level screening proxy, among the milk metabolites, until now only BHB-based tests (strips) have been moderately used to screen dairy cows for SCK directly on the farm [9]. As ketosis is a metabolic disease related primarily to lipid metabolism during the transition period, many lipid molecules of milk are expected to be associated with the onset of this condition. This prompted us to search for lipid biomarkers both at the FA level and across all major lipid classes.

It is known that de novo FAs in milk are synthesized in the mammary gland using acetate (generated by rumen microbial fermentation of plant materials) as the main precursor, but whether the reduced production of C8:0, C10:0, C12:0 and C14:0 in SCK cows is caused by limited supply of precursor remains unclear. Data on dry matter intake and/or blood acetate concentrations for healthy and SCK cows would be required to elucidate this but were not measured in this study. As for the most promising FA biomarker C15:0, it is synthesized by rumen microbes as their structural component (cell membranes) [33]. The underlying mechanism for its severe suppression in the milk of SCK cows remains unclarified. We speculate that reduced dry matter intake in SCK cows, which leads to a massive decline in overall microbial biomass, may be responsible for this suppression. It is interesting to note that the second most promising FA biomarker C18:1t11 is also produced by rumen bacteria (through biohydrogenation) [34]. Our FA profiling results were generally consistent with previous studies. For example, a significant decrease in several de novo (C6:0, C8:0, C10:0, C12:0, C14:0) and medium-chain (C15:0) FAs has previously been observed in the milk of cows with hyperketonemia [35]. However, to the best of our knowledge, this study provides the first evidence identifying milk C18:1t11 as one of the most promising FA biomarkers for SCK.

Among the polar lipids, phospholipids are known to be signalling molecules [19,36], and more likely to be associated with SCK. High-resolution HILIC-MS was thus used to quantify all major phospholipids in milk samples. Chemometric analysis of the LC-MS data matrix revealed a significant shift in the lipidomic profile of milk in the SCK cohort, confirming that the lipidome is influenced by the early stage of the disease. However, the top VIP-scored species identified by the OPLS-DA model were not always consistent across the two years’ milk samples. For example, PI 38:3, PI 38:4 and PI 38:5 were among the top 10 most differentially expressed lipids in 2022, but not in 2023; conversely, PI 30:0, PI 31:0 and PI 32:0 were in the top 10 list in 2023, but not in 2022. This finding is not surprising given that milk lipid composition is a dynamic trait and can be influenced by various factors such as diet, parity and environmental conditions, which were not constant from year to year. Nevertheless, several species from the PC and AcylCar classes were consistently detected as the most differential lipids between control and SCK cows.

Although the top species identified by VIP scores of the OPLS-DA model were statistically the most differential features between the two groups, we also consider the FC in actual lipid abundance an important parameter in biomarker applicability because minor difference, albeit significant, may not be easy to detect with high-throughput but less sensitive techniques (or instruments). Therefore, we combined both the VIP score and the FC in our selection for potential biomarkers. When both *p* value and FC were considered, four lipids (PC 28:0, PC 29:0, PC 30:1 and PC 31:0) stood out as promising biomarkers because these lipids displayed a FC > 2 between the SCK and control groups and met the stringent Bonferroni-corrected significance level (*p* < 0.0004) for two consecutive years. In addition, all these lipids are present in milk at intermediate levels (1–4 µmoles/L) [18], facilitating their measurement for downstream validation and application.

It is worth noting that all four potential phospholipid markers contain odd-chain FA and/or de novo FAs C12:0 or C14:0. For example, PC 29:0 is composed of C14:0 and C15:0 at sn-1 and sn-2 position, whereas PC 28:0 has C12:0-C16:0 or C14:0-C14:0 in its structure [18]. The fact that C15:0, C12:0 and C14:0 were severely reduced in milk of SCK cows suggests that the short supply of these FAs in the mammary gland may be responsible for the reduction in the four phospholipid markers in the SCK group.

Cao et al. [37] reported that deep lipid structural information at both C=C location and *sn*-position could be used to discriminate human cancer tissues from normal tissues. This prompted us to compare the fine structure of lipids between SCK and healthy cows. Using tandem LC-MS and regio-pure standards, we have demonstrated that the FA regio-isomer proportions of PC 34:1 and TAG 52:2 shift significantly in the milk of SCK cows, which could be explored as potential biomarkers for detecting SCK.

The satisfactory performance of the milk lipid feature-based predictive model for samples collected over two years further confirmed that the changing pattern in the milk lipid profile in response to SCK is reproducible and can be largely predicted using milk lipidome data. It is worth mentioning that in this study, control and SCK cows were selected solely based on serum BHB values, and the cut-off threshold of BHB for SCK is often contentious and ranges from 1.2 to 1.4 mmol/L [38,39]. The selection of 1.3 mmol/L as the SCK threshold in this study balances the variation found in the current literature but lacks diagnostic justification. Future studies may combine blood BHB with milk production records and body condition scores for cow grouping, allowing the exclusion of false-positive cases (i.e., BHB > 1.3 mmol/L but otherwise healthy), thereby improving the PLS-DA models. In addition, due to the limited annual incidence of SCK in the study herd, matching samples strictly by DIM was not feasible. However, utilizing a heterogeneous cohort across various DIM stages reflects the real operational variation found at the herd level and the identified milk lipid biomarkers are expected to be applicable across the entire early lactation period. Finally, although our 2022 and 2023 cohorts consisted of different cows and the experiments were conducted over two years, all animals were selected from the same herd and maintained under the same management regime, making it difficult to predict the robustness of the biomarkers and PLS-DA predictive models described in this report.

## 5. Conclusions

Using GC for FA profiling alongside targeted LC-MS lipidomic analysis, we identified two FAs (C15:0 and C18:1t11), and four phospholipids (PC 28:0, PC 29:0, PC 30:1, and PC 31:0), as the most promising milk lipid biomarkers for SCK in dairy cows. PLS-DA predictive models built with milk lipidomic data yielded a cross-year prediction error of 12.5% and pooled data prediction error of 4.5% for healthy and SCK cow classification. Moreover, SCK altered the regio-isomer and double bond isomer composition of milk lipids, which could be explored as additional biomarkers for discriminating SCK cows from healthy cows. Future research will focus on validating the promising biomarkers using herds from different locations or management regimes.

## Figures and Tables

**Table 1:** FA composition of milk from control and SCK cows (% of total FA).

		2022 (*n* = 21)				2023 (*n* = 24)		
FA	Control	SCK	SCK/Control (%)	*p* Value	Control	SCK	SCK/Control (%)	*p* Value
C4:0	2.08	2.43	117	0.000100	1.99	2.30	116	0.000038
C6:0	2.01	1.79	89	0.017643	1.95	1.51	77	0.000000
C8:0	1.55	1.18	76	0.000411	1.48	0.91	61	0.000000
C10:0	3.76	2.49	66	0.000040	3.78	1.82	48	0.000000
C12:0	3.95	2.61	66	0.000054	3.95	1.93	49	0.000000
C14:0	10.80	9.17	85	0.005628	11.50	7.80	68	0.000000
C15:0-iso	0.20	0.19	95	0.084466	0.20	0.18	90	0.089970
C15:0-anteiso	0.46	0.33	72	0.000002	0.43	0.28	65	0.000000
C15:0	1.26	0.68	54	0.000000	1.23	0.61	50	0.000000
C16:0	27.57	27.14	98	0.551632	29.56	27.22	92	0.001321
C16:1c	1.31	1.80	137	0.000014	1.52	2.05	135	0.000002
C17:0-iso	0.58	0.52	90	0.000818	0.51	0.51	100	0.876831
C17:0-anteiso	0.45	0.42	93	0.133877	0.40	0.41	103	0.535171
C17:0	0.83	0.79	95	0.193218	0.77	0.81	105	0.100399
C18:0	13.93	15.91	114	0.003372	12.79	16.50	129	0.000000
C18:1t11	4.35	2.46	57	0.000006	3.43	1.81	53	0.000077
C18:1c9	20.63	26.09	126	0.000067	20.43	29.57	145	0.000000
C18:2n6	2.03	2.04	100	0.916021	2.08	2.10	101	0.799660
C18:3n3	0.88	1.05	119	0.004609	0.90	0.94	104	0.391355
C18:2c9t11	0.93	0.54	58	0.000001	0.75	0.43	57	0.000111

## Data Availability

The original contributions presented in this study are included in the article. Further inquiries can be directed to the corresponding author.

## Abbreviations

The following abbreviations are used in this manuscript:

BHB	β-hydroxy butyrate
SCK	Subclinical ketosis
DIM	Days in milk
FA	Fatty acids
FAME	Fatty acid methyl ester
GC-FID	Gas chromatography–flame ionization detector
LC-MS	Liquid chromatography–mass spectrometry
HILIC	Hydrophilic interaction liquid chromatography
CID	Collision-induced dissociation
OPO	Oleic acid–palmitic acid–oleic acid
OOP	Oleic acid–oleic acid–palmitic acid
CLA	Conjugated linoleic acid
PC	Phosphatidylcholine
PCP	Phosphatidylcholine-plasmalogen
LPC	Lysophosphatidylcholine
PE	Phosphatidylethanolamine
PEP	Phosphatidylethanolamine-plasmalogen
PI	Phosphatidylinositol
PS	Phosphatidylserine
SM	Sphingomyelin
LacCer	Lactosylceramide
AcylCar	Acylcarnitine
PLS-DA	Partial least square-discrimination analysis
VIP	Variable importance in projection
FC	Fold change
